# 4,4′,5,5′-Tetra­methyl-2,2′-[1,1′-(propane-1,3-diyldinitrilo)diethyl­idyne]diphenol

**DOI:** 10.1107/S1600536808027220

**Published:** 2008-08-30

**Authors:** Chin Sing Yeap, Reza Kia, Hoong-Kun Fun

**Affiliations:** a X-ray Crystallography Unit, School of Physics, Universiti Sains Malaysia, 11800 USM, Penang, Malaysia

## Abstract

The title Schiff base compound, C_23_H_30_N_2_O_2_, has crystallographic twofold rotation symmetry. An intra­molecular O—H⋯N hydrogen bond forms a six-membered ring, producing an *S*(6) ring motif. The imino group is coplanar with the benzene ring. The two benzene rings are almost perpendicular to each other, making a dihedral angle of 87.38 (4)°. In the crystal structure, neighbouring mol­ecules are linked along the *c* axis by weak inter­molecular C—H⋯O hydrogen bonds and are further packed into columns along the *b* axis, forming sheets which are parallel to the *bc* plane.

## Related literature

For bond-length data, see: Allen *et al.* (1987 ▶). For hydrogen-bond motifs, see: Bernstein *et al.* (1995 ▶). For information on Schiff base ligands and complexes and their applications, see, for example: Fun, Kargar & Kia (2008 ▶); Fun, Kia & Kargar (2008 ▶); Fun & Kia (2008*a* ▶,*b* ▶,*c* ▶); Calligaris & Randaccio (1987 ▶); Casellato & Vigato (1977 ▶); For a similar structure, see: Fun & Kia (2008*a* ▶).
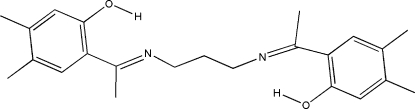


## Experimental

### 

#### Crystal data

C_23_H_30_N_2_O_2_
*M* *_r_* = 366.49
Monoclinic, 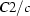

*a* = 28.6398 (12) Å
*b* = 5.1264 (2) Å
*c* = 13.3856 (5) Å
β = 102.090 (5)°
*V* = 1921.67 (13) Å^3^
*Z* = 4
Mo *K*α radiation
μ = 0.08 mm^−1^
*T* = 100.0 (1) K
0.52 × 0.18 × 0.04 mm

#### Data collection

Bruker SMART APEXII CCD area-detector diffractometer
Absorption correction: multi-scan (**SADABS**; Bruker, 2005 ▶) *T*_min_ = 0.959, *T*_max_ = 0.997
22388 measured reflections
2955 independent reflections
2125 reflections with *I* > 2σ(*I*)
*R*_int_ = 0.065

#### Refinement

*R*[*F* ^2^ > 2σ(*F* ^2^)] = 0.061
*wR*(*F* ^2^) = 0.163
*S* = 1.09
2955 reflections
134 parameters
H atoms treated by a mixture of independent and constrained refinement
Δρ_max_ = 0.42 e Å^−3^
Δρ_min_ = −0.23 e Å^−3^

### 

Data collection: *APEX2* (Bruker, 2005 ▶); cell refinement: *APEX2*; data reduction: *SAINT* (Bruker, 2005 ▶); program(s) used to solve structure: *SHELXTL* (Sheldrick, 2008 ▶); program(s) used to refine structure: *SHELXTL* molecular graphics: *SHELXTL*; software used to prepare material for publication: *SHELXTL* and *PLATON* (Spek, 2003 ▶).

## Supplementary Material

Crystal structure: contains datablocks global, I. DOI: 10.1107/S1600536808027220/at2621sup1.cif

Structure factors: contains datablocks I. DOI: 10.1107/S1600536808027220/at2621Isup2.hkl

Additional supplementary materials:  crystallographic information; 3D view; checkCIF report

## Figures and Tables

**Table 1:** Hydrogen-bond geometry (Å, °)

*D*—H⋯*A*	*D*—H	H⋯*A*	*D*⋯*A*	*D*—H⋯*A*
O1—H1*O*1⋯N1	0.94 (3)	1.63 (2)	2.5237 (17)	157 (2)
C12—H12*C*⋯O1^i^	0.96	2.57	3.466 (2)	156

Symmetry code: (i) 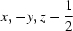.

**Fig. 1.**
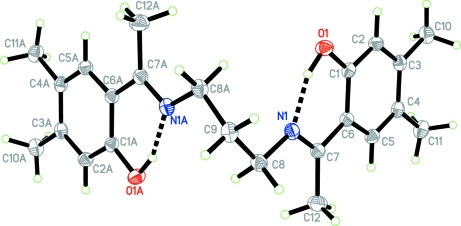
The molecular structure of (I) with atom labels and 50% probability ellipsoids for non-H atoms. The suffix A corresponds to symmetry code (-x + 1, y, -z + 3/2). Intramolecular interactions are shown as dashed lines.

**Fig. 2.**
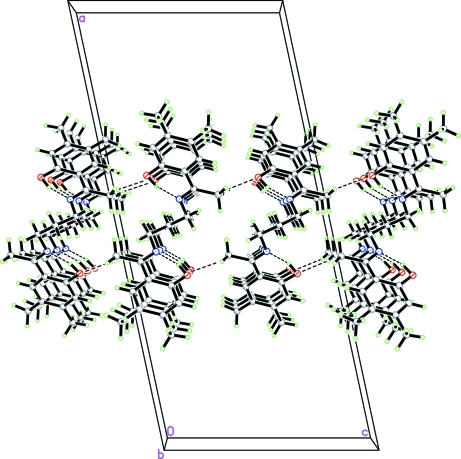
The crystal packing of (I), viewed down the b-axis showing chains along the c-axis and stacking of these chains along the b-axis. Intramolecular and intermolecular interactions are shown as dashed lines.

**Fig. 3.**
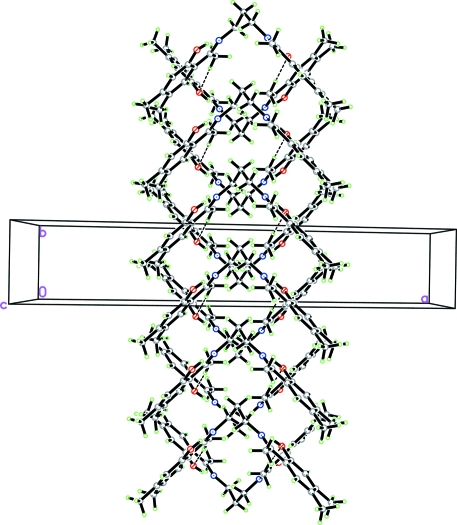
The crystal packing of (I), viewed down the c-axis. Intermolecular interaction are shown as dashed lines.

## supplementary crystallographic information

### Comment

The condensation of primary amines with carbonyl compounds yields Schiff base (Casellato & Vigato, 1977) that are still now regarded as one of the most potential group of chelators for facile preparations of metallo-organic hybrid materials. In the past two decades, the synthesis, structure and properties of Schiff base complexes have stimulated much interest for their noteworthy contributions in single molecule-based magnetism, materials science, catalysis of many reactions like carbonylation, hydroformylation, reduction, oxidation, epoxidation and hydrolysis (Casellato & Vigato, 1977). Only a relatively small number of free Schiff base ligands have been characterized (Calligaris & Randaccio, 1987). As an extension of our work (Fun, Kargar & Kia, 2008; Fun, Kia & Kargar, 2008; Fun & Kia, 2008*a*,*b*,*c*) on the structural characterization of Schiff base ligands and their complexes, the title compound (I), is reported here.

The molecule of the title compound, (I), has a crystallographic twofold rotation symmetry (Fig. 1). The bond lengths and angles are within normal ranges (Allen *et al.*, 1987) and is comparable to its related structure (Fun & Kia 2008*c*). The asymmetric unit of the compound is composed of one-half of the molecule. An intramolecular O—H···N hydrogen bond forms a six-membered ring, producing a *S*(6) ring motif (Bernstein *et al.*, 1995). The imino group is coplanar with the benzene ring. The two benzene rings are almost perpendicular to each other with a dihedral angle of 87.38 (4)°. In the crystal structure, neighbouring molecules are linked together along the *c*-axis  by weak intermolecular C—H···O hydrogen bonds and are further packed into columns along the *b* axis, forming sheets which are parallel to the *bc* plane(Fig. 2, Fig. 3 and Table 1).

### Experimental

The synthetic method has been described earlier (Fun & Kia *et al.*, 2008*c*). Single crystals suitable for *X*-ray diffraction were obtained by evaporation of an ethanol solution at room temperature.

### Refinement

H atom bound to O1 was located from the difference Fourier map and refined freely. The H atom bound to C9 was located from the difference Fourier map and refined freely. The rest of the hydrogen atoms were positioned geometrically and refined using a riding model with C—H = 0.93–0.97 Å and U_iso_(H)= 1.2 U_eq_(C). A rotating-group model was applied for the methyl groups.

### Crystal data

**Table:**

C_23_H_30_N_2_O_2_	*F*_000_ = 792
*M**_r_* = 366.49	*D*_x_ = 1.263 Mg m^−^^3^
Monoclinic, *C*2/*c*	Mo *K*α radiation λ = 0.71073 Å
Hall symbol: -C 2yc	Cell parameters from 2732 reflections
*a* = 28.6398 (12) Å	θ = 3.1–31.1º
*b* = 5.1264 (2) Å	µ = 0.08 mm^−^^1^
*c* = 13.3856 (5) Å	*T* = 100.0 (1) K
β = 102.090 (5)º	Plate, yellow
*V* = 1921.67 (13) Å^3^	0.52 × 0.18 × 0.04 mm
*Z* = 4	

### Data collection

**Table:**

Bruker SMART APEXII CCD area-detector diffractometer	2955 independent reflections
Radiation source: fine-focus sealed tube	2125 reflections with *I* > 2σ(*I*)
Monochromator: graphite	*R*_int_ = 0.065
*T* = 100.0(1) K	θ_max_ = 30.6º
φ and ω scans	θ_min_ = 2.9º
Absorption correction: multi-scan(*SADABS*; Bruker, 2005)	*h* = −40→40
*T*_min_ = 0.959, *T*_max_ = 0.997	*k* = −6→7
22388 measured reflections	*l* = −19→19

### Refinement

**Table:**

Refinement on *F*^2^	Secondary atom site location: difference Fourier map
Least-squares matrix: full	Hydrogen site location: inferred from neighbouring sites
*R*[*F*^2^ > 2σ(*F*^2^)] = 0.061	H atoms treated by a mixture of independent and constrained refinement
*wR*(*F*^2^) = 0.163	*w* = 1/[σ^2^(*F*_o_^2^) + (0.0692*P*)^2^ + 1.4537*P*] where *P* = (*F*_o_^2^ + 2*F*_c_^2^)/3
*S* = 1.09	(Δ/σ)_max_ < 0.001
2955 reflections	Δρ_max_ = 0.43 e Å^−^^3^
134 parameters	Δρ_min_ = −0.23 e Å^−^^3^
Primary atom site location: structure-invariant direct methods	Extinction correction: none

### Special details

**Table:**

Experimental. The low-temperature data was collected with the Oxford Cyrosystem Cobra low-temperature attachment.
Geometry. All esds (except the esd in the dihedral angle between two l.s. planes) are estimated using the full covariance matrix. The cell esds are taken into account individually in the estimation of esds in distances, angles and torsion angles; correlations between esds in cell parameters are only used when they are defined by crystal symmetry. An approximate (isotropic) treatment of cell esds is used for estimating esds involving l.s. planes.
Refinement. Refinement of F^2^ against ALL reflections. The weighted R-factor wR and goodness of fit S are based on F^2^, conventional R-factors R are based on F, with F set to zero for negative F^2^. The threshold expression of F^2^ > 2sigma(F^2^) is used only for calculating R-factors(gt) etc. and is not relevant to the choice of reflections for refinement. R-factors based on F^2^ are statistically about twice as large as those based on F, and R- factors based on ALL data will be even larger.

### Fractional atomic coordinates and isotropic or equivalent isotropic displacement parameters (Å^2^)

**Table:**

	*x*	*y*	*z*	*U*_iso_*/*U*_eq_	
O1	0.39432 (4)	0.1912 (2)	0.79198 (8)	0.0228 (3)	
N1	0.44358 (4)	0.3691 (2)	0.67166 (9)	0.0198 (3)	
C1	0.37375 (5)	0.0311 (3)	0.71524 (10)	0.0186 (3)	
C2	0.33866 (5)	−0.1421 (3)	0.73211 (11)	0.0202 (3)	
H2A	0.3305	−0.1439	0.7958	0.024*	
C3	0.31557 (5)	−0.3111 (3)	0.65766 (11)	0.0196 (3)	
C4	0.32783 (5)	−0.3098 (3)	0.56081 (11)	0.0201 (3)	
C5	0.36250 (5)	−0.1379 (3)	0.54410 (11)	0.0195 (3)	
H5A	0.3705	−0.1376	0.4803	0.023*	
C6	0.38627 (5)	0.0367 (3)	0.61851 (10)	0.0184 (3)	
C7	0.42271 (5)	0.2204 (3)	0.59795 (11)	0.0193 (3)	
C8	0.48072 (5)	0.5502 (3)	0.65557 (11)	0.0220 (3)	
H8A	0.4677	0.6654	0.5991	0.026*	
H8B	0.5067	0.4524	0.6373	0.026*	
C9	0.5000	0.7123 (4)	0.7500	0.0228 (4)	
C10	0.27787 (5)	−0.4945 (3)	0.67857 (12)	0.0248 (3)	
H10A	0.2721	−0.4610	0.7455	0.037*	
H10B	0.2885	−0.6712	0.6750	0.037*	
H10C	0.2489	−0.4686	0.6285	0.037*	
C11	0.30396 (6)	−0.4918 (3)	0.47727 (12)	0.0261 (3)	
H11A	0.3177	−0.4684	0.4183	0.039*	
H11B	0.2704	−0.4542	0.4597	0.039*	
H11C	0.3086	−0.6688	0.5006	0.039*	
C12	0.43456 (6)	0.2315 (4)	0.49364 (12)	0.0292 (4)	
H12A	0.4686	0.2325	0.5005	0.044*	
H12B	0.4213	0.3873	0.4592	0.044*	
H12C	0.4213	0.0818	0.4547	0.044*	
H1O1	0.4165 (8)	0.284 (5)	0.7632 (17)	0.052 (6)*	
H9	0.5269 (6)	0.827 (4)	0.7343 (13)	0.028 (5)*	

### Atomic displacement parameters (Å^2^)

**Table:**

	*U*^11^	*U*^22^	*U*^33^	*U*^12^	*U*^13^	*U*^23^
O1	0.0254 (6)	0.0245 (6)	0.0186 (5)	−0.0057 (4)	0.0047 (4)	−0.0014 (4)
N1	0.0172 (6)	0.0191 (6)	0.0228 (6)	0.0000 (5)	0.0038 (5)	0.0027 (5)
C1	0.0181 (6)	0.0182 (7)	0.0184 (6)	0.0019 (5)	0.0014 (5)	0.0010 (5)
C2	0.0209 (7)	0.0211 (7)	0.0190 (7)	0.0013 (6)	0.0053 (5)	0.0021 (6)
C3	0.0177 (6)	0.0157 (7)	0.0246 (7)	0.0013 (5)	0.0028 (5)	0.0027 (6)
C4	0.0184 (6)	0.0173 (7)	0.0230 (7)	0.0025 (5)	0.0008 (5)	−0.0001 (6)
C5	0.0208 (7)	0.0197 (7)	0.0177 (6)	0.0036 (6)	0.0031 (5)	0.0005 (5)
C6	0.0171 (6)	0.0193 (7)	0.0185 (6)	0.0011 (5)	0.0034 (5)	0.0022 (5)
C7	0.0184 (6)	0.0195 (7)	0.0199 (7)	0.0016 (5)	0.0035 (5)	0.0031 (5)
C8	0.0200 (7)	0.0212 (7)	0.0249 (7)	−0.0008 (6)	0.0050 (6)	0.0037 (6)
C9	0.0192 (10)	0.0193 (11)	0.0297 (11)	0.000	0.0045 (8)	0.000
C10	0.0220 (7)	0.0207 (8)	0.0314 (8)	−0.0012 (6)	0.0050 (6)	0.0022 (6)
C11	0.0266 (8)	0.0222 (8)	0.0271 (8)	0.0004 (6)	−0.0001 (6)	−0.0037 (6)
C12	0.0312 (8)	0.0347 (9)	0.0232 (7)	−0.0063 (7)	0.0094 (6)	−0.0001 (7)

### Geometric parameters (Å, °)

**Table:**

O1—C1	1.3497 (17)	C7—C12	1.505 (2)
O1—H1O1	0.94 (2)	C8—C9	1.5169 (19)
N1—C7	1.2904 (19)	C8—H8A	0.9700
N1—C8	1.4613 (18)	C8—H8B	0.9700
C1—C2	1.395 (2)	C9—C8^i^	1.5169 (19)
C1—C6	1.4143 (19)	C9—H9	1.025 (18)
C2—C3	1.380 (2)	C10—H10A	0.9600
C2—H2A	0.9300	C10—H10B	0.9600
C3—C4	1.412 (2)	C10—H10C	0.9600
C3—C10	1.501 (2)	C11—H11A	0.9600
C4—C5	1.380 (2)	C11—H11B	0.9600
C4—C11	1.506 (2)	C11—H11C	0.9600
C5—C6	1.404 (2)	C12—H12A	0.9600
C5—H5A	0.9300	C12—H12B	0.9600
C6—C7	1.474 (2)	C12—H12C	0.9600
			
C1—O1—H1O1	102.7 (14)	C9—C8—H8A	109.2
C7—N1—C8	119.85 (12)	N1—C8—H8B	109.2
O1—C1—C2	118.58 (12)	C9—C8—H8B	109.2
O1—C1—C6	122.04 (13)	H8A—C8—H8B	107.9
C2—C1—C6	119.38 (13)	C8—C9—C8^i^	113.56 (18)
C3—C2—C1	122.37 (13)	C8—C9—H9	107.5 (10)
C3—C2—H2A	118.8	C8^i^—C9—H9	109.0 (10)
C1—C2—H2A	118.8	C3—C10—H10A	109.5
C2—C3—C4	119.08 (13)	C3—C10—H10B	109.5
C2—C3—C10	120.86 (13)	H10A—C10—H10B	109.5
C4—C3—C10	120.06 (13)	C3—C10—H10C	109.5
C5—C4—C3	118.52 (13)	H10A—C10—H10C	109.5
C5—C4—C11	120.34 (13)	H10B—C10—H10C	109.5
C3—C4—C11	121.14 (13)	C4—C11—H11A	109.5
C4—C5—C6	123.38 (13)	C4—C11—H11B	109.5
C4—C5—H5A	118.3	H11A—C11—H11B	109.5
C6—C5—H5A	118.3	C4—C11—H11C	109.5
C5—C6—C1	117.27 (13)	H11A—C11—H11C	109.5
C5—C6—C7	122.12 (12)	H11B—C11—H11C	109.5
C1—C6—C7	120.61 (13)	C7—C12—H12A	109.5
N1—C7—C6	117.88 (12)	C7—C12—H12B	109.5
N1—C7—C12	121.89 (13)	H12A—C12—H12B	109.5
C6—C7—C12	120.22 (13)	C7—C12—H12C	109.5
N1—C8—C9	111.98 (11)	H12A—C12—H12C	109.5
N1—C8—H8A	109.2	H12B—C12—H12C	109.5
			
O1—C1—C2—C3	179.52 (13)	O1—C1—C6—C5	−179.81 (13)
C6—C1—C2—C3	0.3 (2)	C2—C1—C6—C5	−0.6 (2)
C1—C2—C3—C4	0.1 (2)	O1—C1—C6—C7	−0.1 (2)
C1—C2—C3—C10	−179.85 (13)	C2—C1—C6—C7	179.09 (13)
C2—C3—C4—C5	−0.2 (2)	C8—N1—C7—C6	178.61 (12)
C10—C3—C4—C5	179.74 (13)	C8—N1—C7—C12	−1.6 (2)
C2—C3—C4—C11	179.51 (13)	C5—C6—C7—N1	−178.28 (13)
C10—C3—C4—C11	−0.5 (2)	C1—C6—C7—N1	2.0 (2)
C3—C4—C5—C6	−0.1 (2)	C5—C6—C7—C12	1.9 (2)
C11—C4—C5—C6	−179.86 (13)	C1—C6—C7—C12	−177.83 (14)
C4—C5—C6—C1	0.5 (2)	C7—N1—C8—C9	178.56 (13)
C4—C5—C6—C7	−179.19 (13)	N1—C8—C9—C8^i^	56.33 (9)

Symmetry codes: (i) −*x*+1, *y*, −*z*+3/2.

### Hydrogen-bond geometry (Å, °)

**Table:**

*D*—H···*A*	*D*—H	H···*A*	*D*···*A*	*D*—H···*A*
O1—H1O1···N1	0.94 (3)	1.63 (2)	2.5237 (17)	157 (2)
C12—H12C···O1^ii^	0.96	2.57	3.466 (2)	156

Symmetry codes: (ii) *x*, −*y*, *z*−1/2.
